# (Intending) Migrants and Occupational Downgrading: Investigating the Willingness to Leave the Health Sector Post‐Migration

**DOI:** 10.1002/hpm.70079

**Published:** 2026-04-20

**Authors:** Tunde A. Alabi, Oluwaseun Badru, Fatai A. Badru, Oluwafemi Adeagbo

**Affiliations:** ^1^ Department of Sociology Faculty of Social Sciences University of Lagos Lagos Nigeria; ^2^ Department of Community and Behavioral Health University of Iowa Iowa City Iowa USA; ^3^ Department of Social Work Faculty of Social Sciences University of Lagos Lagos Nigeria

**Keywords:** healthcare workers, japa, migrant health workers, nigerian migrants, professional migrants

## Abstract

The unquenched thirst for emigration in Nigeria‐ and the increasing cost of migrating to the West‐has been established in the literature. Professionals, such as healthcare workers (HCWs), are poorly paid in Nigeria, making many seek better employment abroad. However, many professionals work in less skilled sectors or jobs below their previous position—and education and years of experience—in their home country. While studies have investigated occupational downgrading empirically from the experiences of migrants in the host country, this study focused on HCWs who are still in Nigeria but expressed emigration intentions. This enables us to determine whether migrants are victims of occupational downgrading in the host country or whether it is a deliberate choice. We investigated the willingness among HCWs in Nigeria to accept occupational downgrading post‐migration. Using a cross‐sectional online survey, we analysed responses from 411 respondents who indicated emigration intentions. We computed logistic regression models at a 95% level of significance. We found that 65.3% of healthcare workers reported a willingness to accept occupational downgrading. Male HCWs were twice as likely as their female counterparts to accept occupational downgrading, and pharmacists were 16 times more likely to accept it than medical doctors and dentists. Work hours and job satisfaction were also significantly associated with the acceptance of occupational downgrading. We argue that intending migrants in Nigeria are calculative in their decisions and bracing for occupational downgrading. Nevertheless, they face pressures from disparities between the global South and North in terms of wages, working conditions and quality of life that shape migration decisions and experiences.

## Highlights

- Understanding the willingness of healthcare workers to accept occupational downgrading post‐migration is crucial for effective migration management and staff retention in the health sector.
- Close to two‐thirds of Nigerian healthcare workers who have emigration intentions are willing to leave the health sector post‐migration.
- Factors influencing acceptance of occupational downgrading include gender, job dissatisfaction and being a pharmacist.
- Beyond economic capital, emigration‐induced symbolic and cultural capital can lead professionals in developing countries to willfully emigrate and find jobs in non‐skilled sectors in the West.

## 1 Introduction

International migration remains a topical issue, and its rate is on the increase. The estimated number of international migrants has increased by 87% between 2000 and 2024, resulting in an increase from 150 million to 281 million migrants [1]. In an attempt to answer the question of why people migrate, Carling [2] provided eight reasons, including encountering an opportunity to migrate, choice or force, the intrinsic and instrumental value of migration, and ‘to lead a normal life’ (p. 1774). Similarly, de Haas [3] utilised the agency‐structure approach to explain the migration process. Both scholars expanded migration theories beyond economic determinations to capture broader personal, political and social forces shaping migration [2, 3].

While migration is not a problem, the challenges faced by migrants in the host country, particularly concerning securing a befitting job and continuing their line of profession and transitioning into new roles in the host country, have been noted in several countries, including South Africa [4], Australia [5, 6, 7, 8], Canada [9, 10], United States and United Kingdom [11, 12, 13]. The most affected are usually migrants from developing countries who have migrated to developed economies. This leads to occupational downgrading, a situation in which migrants are unable to find jobs that match their qualifications and previous experience in their home country [11, 13]. Despite these employment challenges, the international migration rate, particularly from Africa to the West, has continued to rise, underscoring the need to consider factors beyond economics.

In Nigeria, the growing popularity of emigration as a means of escaping the uncertainty of the country's political and socio‐economic landscape is not in doubt [14]. For many Nigerians, living in the West indicates supremacy and a better life [12] and signifies social, symbolic and cultural capital [15]. From the perspective of Nigeria and some African countries, people may migrate to improve their social status and that of their family members at home [12, 16]. The deference to returning migrants and the popularity of the phrase: “I just got back” (IJGB), especially during December breaks, lends credence to the migration‐status nexus and Bourdieu's theory of capital. Consequently, many Nigerians are eager to migrate by all means possible, including those who are gainfully employed [17, 18, 19]. Those who cannot afford the high cost of migration resort to borrowing and selling properties [20].

In recent times, the outflow of healthcare workers (HCWs) from Nigeria has attracted public attention [17, 21, 22, 23]. This is to the extent that the country's national assembly in 2023 attempted to mandate Nigeria‐trained medical doctors to practice in Nigeria for at least five years before leaving the country, which generated public debates and backlash [24]. Some scholars have documented the effect of HCWs' emigration on brain drain in the health sector and the resultant poor quality of healthcare delivery [25]. However, it is worth noting that the migration of HCWs from low‐income to high‐income countries has always existed [26]. In addition, HCWs migrate from one high‐income country to another where the pay is substantially higher [27].

In clear terms, migration for economic, political, and social reasons is a longstanding phenomenon that benefits both receiving and sending countries. However, the career trajectory of migrants, especially HCWs, in the host country is worthy of attention. While the emigration of HCWs is a colossal loss for Nigeria, it is a brain waste for the professional migrants who leave their profession post‐migration, considering (1) the extended number of years required to study health‐related courses, including training, and (2) the amount of previous experience in the home country, which may go to waste. In addition, occupational downgrading leads to the underutilisation of human resources [28], especially in the health sector, where human resources are already scarce. It makes migrants question their professional identity [13].

Some studies have identified factors related to migrants' experience of occupational downgrading. One of these factors includes structural barriers. One structural barrier outside the reach of the immigrants is references [29]. Employers in the host country often prefer applicants to have local referees (i.e., referees who are citizens of the host country). This may be difficult for newly arrived immigrants who may not know nationals who can serve as their referees. A second factor which majorly affects highly skilled immigrants is occupational licencing. Some studies investigated the role of occupational licencing on immigrants' employment trajectory in Canada [30, 31]. Skilled migrants (such as doctors, nurses, lawyers, engineers, surveyors, etc.) in their country of origin are expected to be licenced in the host country before they can practise. In some instances, the procedures to become licenced are herculean. Hence, professional migrants experience occupational downgrading when they migrate to another country where the same profession is highly regulated [30, 31]. The non‐recognition of foreign certificates and skills of migrants has also been reported in Australia [6, 7] and the United States [32]. Due to regulations, non‐nationals interested in such professions must undergo re‐examination and comply with the regulatory frameworks of the host country before receiving authorisation to work. In the case of migrant nurses in Norway, Korzeniewska and Erdal [28, p. 14] noted thus:

There are structural issues, such as authorisation (or not) and (lack of) permission to work in Norway as a nurse…and there is the degree to which nurses' highly specialised skills are (recognized and) utilised (or not) in their current workplaces…some nurses resign from further pursuit of authorisation and simply take up work outside of their profession.

Third, systematic bias and sentiments against certain nationalities in the host country may influence migrants' experiences of occupational downgrading [7, 33].

Aside from structural factors, migrants' socio‐demographic characteristics can also affect migrants' employment and occupational downgrading in the host country. Some studies have identified the influence of names [8, 33]. Half of the participants of migrants in the study of Ryndermann and Flynn [8] cited their indigenous names as the possible reasons for their experience of occupational downgrading. In the study of Udah et al. [33], a participant narrated that writing applications and you put your name, and it sounds African, you will be judged on that… I have heard a friend who said to me, ‘the best thing is to change the name and put another name’ [33, pp. 66–67]. Some Nigerians have taken to social media to lament being rejected by employers until they change their first name to an English one. Changing names can increase migrants' chances of being called for interviews, but it does not guarantee securing a job [34]. Some studies have documented that male professional migrants (such as engineers) are more likely to continue working in their line of profession than their female counterparts [35]. Migrants' religion may also affect their chances of experiencing occupational downgrading, and Muslims are more likely to be disadvantaged, as explained by the concept of the ‘Muslim penalty’ [36].

Some studies have identified how work conditions in the home country may contribute to workers' desire to emigrate. In the health sector, poor work conditions, including the experience of burnout, personnel shortage and overworking of employees, fatigue, lack of adequate equipment, job dissatisfaction and inadequate compensation have been identified as factors contributing to the exodus of Nigerian HCWs [17, 23, 26, 37, 38, 39]. However, these studies investigated emigration intentions among HCWs and did not specifically focus on willingness to accept occupational downgrading post‐migration.

Our study contributes to research and fills knowledge gaps in at least two ways. First, it highlights the potential influence of the willingness to accept occupational downgrading and how it varies across different healthcare professions, which is crucial for professional identity [13]. Second, existing studies on occupational downgrading focused on the experiences of people who have already migrated [5, 6, 7, 8, 11, 13, 40]. This study focused on HCWs who are still in Nigeria but intend to emigrate. Hence, the current study provides insight into migrants' agency in occupational downgrading.

This study examines the willingness to accept occupational downgrading after emigration, as well as the associated demographic and work‐related factors. We investigated the proportion of HCWs willing to leave the health sector post‐migration and the possible influence of demographic characteristics (gender, age, education, religion, marital status, ethnicity and region of birth) and work‐related characteristics, such as profession, region of medical practice, type of health facility, experience of burnout, duration of practice, work hours and job satisfaction, on the acceptance of occupational downgrading. An understanding of the factors associated with the willingness to accept occupational downgrading post‐migration can help policymakers in Nigeria develop strategies to reduce brain drain by improving work conditions. It can also inform governments of major destination countries about the need for a stronger and more effective skill‐recognition framework for migrant HCWs.

## 2 Theoretical Framework

Occupational downgrading explains situations where migrants—after arriving in the host country—are unable to find jobs that befit their educational attainment and previous job experience in their host country, thereby resorting to employment in another sector or lower jobs within the same sector [8, 11, 13, 28]. To (Adversario [11, p. 6]), occupational downgrading ‘happens when immigrants hold lower‐level jobs upon migration than their last occupations from their countries of origin’. Highly educated and professional migrants, including HCWs, are more likely to experience occupational downgrading. Studies have documented the experiences of professional migrants with occupational downgrading. Rynderman and Flynn [8] described situations in which educated and skilled migrants with years of experience in their country of origin resorted to unskilled jobs such as driving and cleaning in the host country. Alabi [12] discussed how Nigerian migrants with postgraduate degrees and years of experience in Nigeria resorted to care work in the US and UK. The negative impacts of occupational downgrading on the mental health of migrants have been documented [41, 42]. In addition, the stigma associated with return migration without substantial achievement may force migrants to remain in the host country despite the detrimental effects of occupational downgrading.

However, at least two critical issues and questions relate to occupational downgrading. First, one wonders whether migrants are (un)aware of the possibility of occupational downgrading abroad. In the case of Nigerian migrants, Alabi [12] found that many migrants had exaggerated views of life in the West before emigration—they thought they would continue in the same line of profession and experience upward occupational mobility. However, despite the disappointments experienced by Nigerian migrants, those at home want to migrate, too, at all costs, irrespective of the availability of job opportunities or restrictions in the host country [6, 12, 14]. Migrants' experiences of occupational downgrading in the host countries have not significantly deterred the aspirations of Nigerians at home. Bourdieu's forms of capital help to understand that, beyond the economic capital migrants gain, symbolic and cultural capital are important to understanding emigration intentions and the willingness to accept occupational downgrading post‐migration [15]. In Nigeria, migrants are revered and believed to have cultural capital. Their ways of communicating, dressing and eating are considered to be modern. They are believed to have a legitimate culture, and their families at home are respected regardless of the status and conditions of migrants in their host country. An average Nigerian would prefer an imported commodity to an indigenous one. Having a PhD from a foreign university, especially from the West, is an honour and respected than studying in Nigeria. Nigerian families who have relatives abroad are accorded public respect. The honour and respect migrants and their families enjoy have been described in an earlier study as ‘migration‐facilitating capital’ [43].

By implication, one may express caution in putting all the blame for occupational downgrading on structural factors in the host countries. Migrants could have calculated their risks and benefits and were willing to accept occupational downgrading before emigration. A few examples may suffice here. There is evidence that earning power and exchange rates are connected to migration and that people from countries with weak currencies migrate to countries with strong currencies, all things being equal [44]. One US dollar is equivalent to about 1400 Nigerian Naira as of April 2026. Nigerian skilled workers and professionals, including healthcare workers and academics, earn less than 800 US dollars monthly. Consequently, a postdoctoral student, taxi driver, care worker, mason, etc., in the West can earn more than a professional living and working in Nigeria. Hence, there are instances of Nigerians with postgraduate degrees migrating to the UK via the student visa route to study—for degrees they already have—and working 20 h per week. The logic is that (1) what they earn from working 20 h per week in the UK earns them more money than a ‘professional career’ in Nigeria, and (2) the UK's graduate visa allows students to stay for 2 years and work for unlimited hours in the UK after completing their study.

The argument here is that migrants might be aware and willingly accept occupational downgrading before emigration, which is an essential contribution to this study— it focuses on the willingness to accept occupational downgrading post‐migration among HCWs currently in Nigeria. Despite people's agency, their migration decisions are shaped by the global north‐south disparities in development indicators, including wages, currency value and quality of life. The global disparities also shape migration‐facilitating capital. Hence, buffers and different forms of capital motivate migrants (particularly Africans in the West) to remain in the host country and encourage those at home to emigrate. Many skilled migrants who experience occupational downgrading and work in unskilled sectors still earn more and send money to their relatives at home. In addition, their status as international migrants is symbolic and confers cultural capital, as they are viewed as holders of legitimate culture.

## 3 Methods

### 3.1 Study Design, Study Setting, and Population

We conducted a cross‐sectional online survey in Nigeria. The comprehensive method was published elsewhere [22]. In brief, with a pretested questionnaire, we surveyed HCWs in all 36 states in Nigeria and the Federal Capital Territory to understand their migration intention. We collected data between May and June 2023.

### 3.2 Data Collection and Sampling Technique

The total sample size was 513. In this study, however, we focused on the 411 who expressed the intention to emigrate in line with our research objective. We sent a Survey Monkey link to HCWs via social media, including Twitter, Facebook, Telegram, and WhatsApp (including HCWs' specific organizational bodies). We encouraged HCWs who were at least 18 years old and lived in Nigeria to complete the survey. The internet protocol address provided by Survey Monkey was used to identify and exclude those who completed the survey from outside Nigeria. The study was approved by the Sokoto State Ministry of Health (SKHREC/019/2023).

### 3.3 Measures

The variable of interest was occupational downgrading. We asked HCWs if they were willing to leave the health sector post‐migration: ‘There are cases of medical personnel who were unable to find jobs in the medical sector or continue with the profession they were practising in Nigeria. When you migrate, are you willing to work in other sectors if you are unable to find a job in the medical sector?’ We considered HCWs who responded yes to be willing to engage in occupational downscaling [22].

The independent variables include demographic factors, work‐related characteristics, job satisfaction, and burnout.

#### 3.3.1 Sociodemographic Characteristics

We obtained HCWs' age, sex, education, ethnic group, where they were born within Nigeria, religion, marital status, and where they received their bachelor's degrees.

#### 3.3.2 Employment and Work‐Related Characteristics

Profession, employment status, work setting, length of practice, and work hours per day.

#### 3.3.3 Job Satisfaction

We asked whether they were satisfied with their jobs using a single‐item modified Job Satisfaction Scale on a 7‐point Likert scale (extremely dissatisfied = 1 to extremely satisfied = 7). We considered HCWs who endorsed options with dissatisfaction to be dissatisfied with their jobs [45].

#### 3.3.4 Burnout

We assessed if they experienced burnout with a validated single‐item Maslach **Burnout** Inventory Scale on a 7‐point Likert scale (never = 0 to every day = 6). We classified HCWs who endorsed once a week or more to experience burnout [46, 47].

### 3.4 Data Analysis

We analysed data on SPSS version 29. We used frequency and percentage to analyse categorical variables. We ran a 2‐by‐2 analysis and reported the frequency and percentages but used the bivariate binary regression analysis to determine explanatory variables associated with occupational downgrading at *p* 0.25. These variables were subjected to the Variance Inflation Test to assess for collinearity. We found no evidence of multicollinearity. Then, we modelled these variables in a multivariate logistic regression. We considered variables significant at a 95% confidence interval to influence occupational downscaling among the HCWs.

## 4 Results

### 4.1 Demographic Characteristics

Table 1 shows that most HCWs (41.8%) were in their thirties. Less than 10% of the respondents had attained age 50. The mean age was 38.1, with a standard deviation of 10.01. More than half (55%) of the sample was female. More than half (59.9%) of the respondents had a bachelor's degree; 17.8% had master's degrees. Interestingly, a little less than three‐quarters (74.1%) attended federal universities in Nigeria for their first degree. In Nigeria, federal universities are cheaper and more affordable than state and private universities. About 5% attended private universities, while 13.8% attended state universities.

**TABLE 1:** Sociodemographic characteristics (*N* = 411).

Variables	*n*	(%)
Age (years)^a^		
20–29	100	24.6
30–39	170	41.8
40–49	100	24.6
50 and above	37	9.0
Sex		
Female	226	55.0
Male	185	45.0
Education^a^		
Bachelor	241	58.9
Diploma	25	6.1
Masters	73	17.8
Residency	47	11.5
Fellowship/PhD	23	5.7
First‐degree location		
Federal university in Nigeria	295	74.1
State university in Nigeria	55	13.8
Private nigerian university	19	4.8
University in other parts of Africa	11	2.8
University outside Africa	18	4.5
Profession		
Medical doctor/Dentist	128	31.1
Nurse	122	29.7
Physiotherapist/Occupational therapist	81	19.7
Pharmacist	34	8.3
Medical lab scientist	20	4.9
Others	26	6.3
Marital status		
Single/Never married	127	30.9
Married/Cohabiting	270	65.7
Separated/Divorced/Widowed	14	3.4
Ethnicity^a^		
Yoruba	180	43.9
Igbo	63	15.4
Hausa	66	16.1
Others	101	24.6
Religion^a^		
Islam	123	30.1
Christianity	286	69.9
Region born in^a^		
North	158	38.6
South	251	61.4

^a^ Signifies variables less than 411 count.

The sample comprised 31.1% of medical doctors and dentists, 29.7% of nurses, 19.7% of physiotherapists or occupational therapists, 8.3% of pharmacists, 4.9% of medical lab scientists, and 6.3% of healthcare workers in other categories, which included radiographers, optometrists, nutritionists, etc. Most (65.7%) respondents were married or cohabiting. About 44% of the respondents were Yoruba‐an ethnic group domiciled in the south‐western part of the country‐, 16.1% were Hausa‐domiciled in the northern region‐, while 15.4% were Igbo‐the major ethnic group in the south‐eastern part of the country. Approximately 61% of the sample said they were born in the country's southern region, while 38.6% were born in the north. However, as displayed in Table 2, 63.8% of the respondents were practicing in the Southern region as of the time of data collection, while 36.2% were practicing in the North.

**TABLE 2:** Employment and work‐related characteristics of the HCWs.

Variables	*n*	(%)
Employment status		
Unemployed	36	8.8
Employed	375	91.2
Region of current practice^a^		
North	138	36.2
South	243	63.8
Work setting		
Tertiary	209	54.0
Secondary	58	15.0
Primary	18	4.7
Private facility	50	12.9
Non‐governmental organisation/Others	52	13.4
Burnout		
Yes	205	49.9
No	206	50.1
Duration of practice (years)^a^		
1–5	132	32.6
6–10	111	27.4
11–15	76	18.8
16–20	43	10.6
20 and above	43	10.6
Work hours per day (hours)^a^		
1–8	229	57.1
Above 8	172	42.9
Job satisfaction		
Satisfied	231	56.2
Not satisfied	180	43.8

^a^ Signifies variables less than 411 count.

### 4.2 Work‐Related Characteristics

An overwhelming majority (91.2%) of the respondents were employed. More than half (54%) of the respondents were practicing at tertiary facilities, compared to 15% at secondary facilities and 4.7% at primary facilities. Over a quarter (26.3%) worked at a private facility or with a non‐governmental organisation (NGO). The lower number of respondents working at the primary and secondary facilities is not surprizing. In Nigeria, all things being equal, lower‐level facilities are less likely to be funded and equipped than those at the higher levels, despite the former being the primary point of contact for many healthcare seekers. Close to a third (32.7%) of the respondents who worked with NGOs were nurses; 21.2% were pharmacists, while 19.2% were medical doctors. Most (61.1%) of those working in primary facilities were medical doctors.

The average duration of practice was 12 years ± 9.45. More than half (57.1%) of the HCWs said they worked 1–8 h daily, while 42.9% said they worked more than 8 hours. Approximately 41% of those who reported working above 8 hours daily were medical doctors; 35.5% were nurses. Approximately half of the respondents reported burnout at their jobs. More than half of the nurses (54.9%) reported burnout compared to 48.4% of doctors, 44.9% of pharmacists, and 40% of medical lab scientists. Interestingly, more than half (56.2%) of the respondents reported being satisfied with their jobs. Three‐quarters of the medical laboratory scientists reported job satisfaction compared to 55.9% of pharmacists and 55.7% of nurses. Medical doctors were the least satisfied with their jobs (42.2%).

### 4.3 Willingness to Accept Occupational Downgrading and Associated Factors

We found that 65.3% of the respondents were willing to accept occupational downgrading after migration. Table 3 shows the factors associated with occupational downgrading at the bivariate level.

**TABLE 3:** Bivariate analysis between explanatory variables and occupational downgrading.

Variables	Occupational downgrading		Crude odds ratio	95% CI	*p*‐value
	No (%)	Yes (%)			
Age (years)					
20–29	27 (28.1)	69 (71.9)	1		
30–39	50 (31.8)	107 (68.2)	0.837	0.480–1.462	0.533
40–49	37 (38.9)	58 (61.1)	0.613	0.334–1.125	0.114
50 and above	19 (51.4)	18 (48.6)	0.371	0.169–0.811	0.013
Sex					
Female	90 (42.1)	124 (57.9)	1		
Male	45 (25.7)	130 (74.3)	2.097	1.358–3.237	< 0.001
Education					
Bachelor	67 (29.4)	161 (70.6)	1		
Diploma	10 (41.7)	14 (58.3)	0.583	0.247–1.377	0.218
Masters	27 (39.7)	41 (60.3)	0.632	0.360–1.110	0.110
Residency	19 (42.2)	26 (57.8)	0.569	0.295–1.098	0.093
Fellowship/PhD	12 (54.5)	10 (45.5)	0347	0.143–0.841	0.019
First degree location					
Federal university in Nigeria	100 (35.8)	179 (64.2)	1		
State university in Nigeria	22 (44.0)	28 (56.0)	0.711	0.386–1.308	0.273
Private nigerian university	5 (26.3)	14 (73.7)	1.564	0.547–4.470	0.404
University in other parts of Africa	2 (18.2)	9 (81.8)	2.514	0.533–11.863	0.244
University outside Africa	3 (16.7)	15 (83.3)	2.793	0.790–9.882	0.111
Profession					
Medical doctor/Dentist	46 (38.0)	75 (62.0)	1		
Nurse	56 (48.3)	60 (51.7)	0.657	0.392–1.102	0.111
Physiotherapist/Occupational therapist	23 (29.9)	54 (70.1)	1.440	0.782–2.652	0.242
Pharmacist	2 (6.1)	31 (93.9)	9.507	2.172–41.607	0.003
Medical lab scientist	3 (15.8)	16 (84.2)	3.271	0.904–11.83	0.071
Others	5 (21.7)	18 (78.3)	2.208	0.768–6.352	0.142
Marital status					
Single/Never married	33 (27.0)	89 (73.0)	1		
Married/Cohabiting	98 (38.6)	156 (61.4)	0.590	0.368–0.947	0.029
Separated/Divorced/Widowed	4 (30.8)	9 (69.2)	0.834	0.241–2.894	0.775
Ethnicity					
Yoruba	70 (41.2)	100 (58.8)	1		
Igbo	16 (26.7)	44 (73.3)	1.925	1.006–3.682	0.048
Hausa	15 (24.2)	47 (75.8)	2.193	1.137–4.229	0.019
Others	33 (34.4)	63 (65.6)	1.336	0.794–2.248	0.275
Religion					
Islam	39 (33.3)	78 (66.7)	1		
Christianity	95 (35.2)	175 (64.8)	0.921	0.582–1.457	0.725
Region born in					
North	42 (27.5)	111 (72.5)	1		
South	93 (39.6)	142 (60.4)	0.578	0.372–0.898	0.015
Employment status					
Unemployed	7 (20.6)	27 (79.4)	1		
Employed	128 (36.1)	227 (63.9)	0.460	0.195–1.086	0.076
Region of current practice					
North	38 (29.0)	93 (71.0)	1		
South	90 (39.3)	139 (60.7)	0.631	0.398–1.001	0.050
Work setting					
Tertiary	74 (37.4)	124 (62.6)	1		
Secondary	20 (37.0)	34 (63.0)	1.015	0.544–1.891	0.964
Primary	8 (44.4)	10 (55.6)	0.746	0.282–1.974	0.555
Private facility	11 (23.4)	36 (76.6)	1.953	0.937–4.069	0.074
Non‐governmental organisation/Others	17 (34.7)	32 (65.3)	1.123	0.584–2.162	0.728
Burnout					
Yes	67 (34.4)	128 (65.6)	1		
No	68 (351)	126 (64.9)	0.970	0.639–1.472	0.886
Duration of practice (years)					
1–5	33 (26.0)	94 (74.0)	1		
6–10	35 (34.3)	67 (5.7)	0.672	0.380–1.188	0.171
11–15	29 (40.8)	42 (59.2)	0.508	0.274–0.943	0.032
16–20	14 (34.1)	27 (65.9)	0.677	0.317–1.444	0.313
20 and above	21 (50.0)	21 (50.0)	0.351	0.170–0.724	0.005
Work hours per day (hours)					
1–8	68 (31.8)	146 (68.2)	1		
Above 8	62 (37.6)	103 (62.4)	0.774	0.505–1.186	0.239
Job satisfaction					
Satisfied	86 (38.6)	137 (61.4)	1		
Not satisfied	49 (29.5)	117 (70.5)	1.499	0.976–2.302	0.064

Approximately 72% of respondents aged 20–29 expressed willingness to accept occupational downgrading; the figures are 68.2% for those aged 30–39, 61.1% for the 40–49 group, and 48.6% for those aged 50% and above. Male healthcare workers were significantly more likely to accept occupational downgrading than their female counterparts (74.3% vs. 57.9%, *p* < 0.001, OR: 2.1). The most willing to accept occupational downgrading were those with Bachelor's degree (70.6%), while the least willing were people with fellowship/PhD (45.5%) (*p*: 0.019). Compared to medical doctors/dentists, pharmacists were 9.5 times more likely to accept occupational downgrading‐ 93.9% of them indicated that they are willing to work in non‐health sectors after migration. The least willing to accept occupational downgrading was nurses (51.7%), followed by medical doctors/dentists (62%). Compared to single HCWs, the married ones were less likely to accept occupational downgrading (OR: 0.590, *p*: 0.029). Interestingly, ethnicity showed some associations. Compared to Yoruba HCWs, the Igbo were 1.9 times more likely to accept occupational downgrading (*p*: 0.048), while Hausa were 0.2 times more likely (*p*: 0.019). The region in which HCWs were born and where they currently practice were associated with willingness to accept occupational downgrading. Those born in the South had lower odds of accepting occupational downgrading compared to those born in the North (OR: 0.578, *p*: 0.015). Similar results were obtained for the region of current practice (OR: 0.631, *p*: 0.050). Surprisingly, work setting (primary facility, secondary facility, etc.), experience of burnout, number of working hours, and job satisfaction were not significantly associated with willingness to accept occupational downgrading at 95%. However, the duration of practice showed an interesting but expected association. Compared to those who have practiced for 1–5 years, HCWs who have practiced for 20 years or more had lower odds of accepting occupational downgrading (OR: 0.351, *p*: 0.005).

Table 4 shows the adjusted effects of the factors in the same model. Four variables were significantly associated with the acceptance of occupational downgrading. Two variables (gender and professional) are socio‐demographic factors and were also significant at the bivariate level. The two other factors (work hours and job satisfaction) are work‐related factors and were not significant at the bivariate or crude level.

**TABLE 4:** Factors associated with occupational downscaling.

Variables	Adjusted odds ratio	95%	CI	*p*‐value
Age (years)				
20–29	1			
30–39	1.050	0.382	2.889	0.924
40–49	1.249	0.325	4.806	0.746
50 and above	0.514	0.078	3.394	0.489
Sex				
Female	1			
Male	2.004	1.008	3.983	**0.047**
Education				
Bachelor	1			
Diploma	1.020	0.291	3.576	0.976
Masters	0.544	0.259	1.142	0.108
Residency	0.408	0.152	1.094	0.075
Fellowship/PhD	0.305	0.092	1.011	0.052
First degree location				
Federal university in Nigeria	1			
State university in Nigeria	1.006	0.483	2.095	0.988
Private nigerian university	2.642	0.788	8.861	0.116
University in other part of Africa	2.685	0.464	15.533	0.270
University outside Africa	3.965	0.782	20.094	0.096
Profession				
Medical doctor/Dentist	1			
Nurse	0.586	0.239	1.435	0.242
Physiotherapist/Occupational therapist	0.996	0.378	2.626	0.993
Pharmacist	15.983	3.093	82.584	**0.001**
Medical lab scientist	1.564	0.334	7.328	0.570
Others	1.335	0.348	5.129	0.674
Marital status				
Single/Never married	1			
Married/Cohabiting	1.076	0.474	2.441	0.861
Separated/Divorced/Widowed	2.566	0.482	13.659	0.269
Ethnicity				
Yoruba	1			
Igbo	1.761	0.789	3.929	0.167
Hausa	0.734	0.222	2.425	0.612
Others	0.558	0.244	1.278	0.168
Region born in				
North	1			
South	0.798	0.323	1.969	0.624
Employment status				
Unemployed	1			
Employed	0.440	0.047	4.137	0.472
Region of current practice				
North	1			
South	0.912	0.375	2.222	0.840
Work setting				
Tertiary	1			
Secondary	0.848	0.372	1.933	0.695
Primary	0.949	0.267	3.376	0.936
Private facility	0.997	0.383	2.599	0.996
Non‐governmental organizations/Others	0.690	0.294	1.622	0.395
Duration of practice (years)				
1–5	1			
6–10	0.853	0.318	2.291	0.753
11–15	0.661	0.198	2.208	0.501
16–20	1.340	0.318	5.653	0.690
20 and above	1.167	0.184	7.408	0.870
Work hours per day (hours)				
1–8	1			
Above 8	0.539	0.304	0.955	**0.034**
Job satisfaction				
Satisfied	1			
Not satisfied	2.458	1.399	4.318	**0.002**

*Note:* Bolded *p*‐value are significant; Nagelkerke *R* ^2^: 0.264.

Male HCWs were twice as willing to accept occupational downgrading than their female counterparts (*p*: 0.047). Pharmacists were approximately 16 times more likely than medical doctors/dentists to accept occupational downgrading (*p* < 0.001). Medical doctors/dentists significantly differed from other healthcare workers in accepting occupational downgrading. Surprisingly, HCWs who worked for 8 hours per day were less likely to accept occupational downgrading than those who worked less than 8 hours (OR: 0.539, *p*: 0.034). HCWs unsatisfied with their jobs were 2.5 times more likely to accept occupational downgrading than those who reported job satisfaction.

## 5 Discussion and Limitations

The study investigated the willingness to accept occupational downgrading among HCWs currently living in Nigeria and expressed the intention to emigrate. We found that six in 10 HCWs who intend to emigrate were willing to accept occupational downgrading; that is, they are willing to work in non‐health sectors. Before we provide probable explanations for a high willingness to accept occupational downgrading, it is essential to first explain factors associated with its acceptance.

This study found that male HCWs were more willing to accept occupational downgrading than their female counterparts. Ironically, earlier studies found that female migrants were more likely to experience employment challenges in the host country [35]. However, the current study focuses on willingness rather than actual experience. A probable explanation for why male HCWs are more willing to accept occupational downgrading than females is the societal expectations of different genders. The idea of being a man in Nigeria is synonymous with the ability to provide financially. Hence, men are more pressured to do any work to earn money than women.

This study also found that pharmacists were 16 times as likely as medical doctors and dentists to be willing to accept occupational downgrading. While this is interesting, a future qualitative study is required to provide adequate explanations for this finding. Although Alabi [34] found that the experience of occupational downgrading and willingness to accept it are expected to vary by course of study and skills, with those in the humanities at a disadvantage, they did not report variations within the medical fields. One logical reason is that medical doctors and dentists typically spend more years in training than pharmacists; hence, the former are unwilling to ‘settle for less’. In addition, medical doctors tend to be more respected than community pharmacists, as the former is believed to determine the nature of the conversation between patients and the latter. Furthermore, medical doctors are in higher demand than pharmacists. Hence, the idea of professional identity and how HCWs identify with their jobs is essential [13, 28].

The finding that HCWs who were unsatisfied with their jobs were more likely to accept occupational downgrading than those who reported job satisfaction is expected and common‐sensical. It resonates with earlier studies on the nexus between job satisfaction and migration intention among HCWs in Nigeria and elsewhere [22, 39, 48]. Job dissatisfaction may signal poor working conditions, including wages, structural facilities, and medical equipment, in Nigeria's health sector to the extent that HCWs are willing to leave the sector after migration. Positive work conditions are expected to improve affective commitment, which is defined as employees' emotional attachment to their work and organisation [49]. While employees who have affective commitment may remain in the organisation and line of job, those who are dissatisfied may be interested in either leaving the organisation and or leaving the job or country completely to start afresh elsewhere.

We return to the high prevalence of willingness to accept occupational downgrading after migration. It is important to note that health‐related disciplines have the longest study duration in Nigeria. The study duration is 6 years for medicine and surgery, and dentistry; it is 5 years for other health courses such as pharmacy, nursing, physiotherapy, etc. Graduands spend 1 year on housemanship or internship and an additional year for the national youth service, making a total of at least 8 years (to become medical doctors or dentists) and 7 years for other HCWs. The intense competition for health courses at top public universities warrants further analysis. Despite these years of investment, why would they accept occupational downgrading? There are a few probable explanations.

First, the desperation to emigrate from Nigeria is supported by the assertions and findings from earlier studies [6, 12, 14, 17, 18, 19, 20]. Affirming this point, (Liu [14, p. 8]) reported that emigration “is a visceral response to youth's refusal to cope with extant temporal distortions, whereby their overwhelming frustrations reframe migration as a matter of survival rather than choice.” To many young and middle‐class people, there is no hope in sight, and leaving Nigeria is the only way for them to secure a better future. They are willing to accept any job in a society that is perceived as sane and stable. Consequently, the three most preferred destination countries selected by the respondents in this study are the UK (26.5%), Canada (22.4%), and the US (20%). The increasing inflation—without a corresponding increase in earnings and Naira's devaluation might have also fuelled the desperation to emigrate. Hence, judging from the ages and years of experience, most of the respondents in the study earned less than $1000. There are instances where medical doctors in Nigeria work as Uber/Bolt drivers to supplement their earnings. Meanwhile, less‐skilled workers in the US, UK, and Canada earn more, and this reinforces the role of exchange rates in migration decisions [44].

The second point, related to the first, is the need to “to lead a normal life” [2, p. 1774]. To a non‐Nigerian, the second point may appear ordinary, but experiencing what life is like in Nigeria and in the West will show that (ab)normal life is more than enough reason for HCWs to want to emigrate and accept occupational downgrading. It is important to wonder what constitutes (ab)normal life. Drawing on qualitative studies of migrants in Bolivia and Poland, Carling [2] noted the ability to afford basic necessities (including holidays) without worry or resorting to borrowing. In Nigeria, however, the reality is different. Alabi [12] noted that Nigerian migrants in South Africa appreciated “load‐shedding,” in which electricity is interrupted for a couple of hours. A timetable shows when electricity will be interrupted and restored in different geographical areas. To such Nigerian migrants, this constituted a normal life because, in Nigeria, power could be interrupted without prior notice and no explanation regarding when it would be restored. In November 2024, relatives and friends of patients at the foremost University College Hospital, Ibadan, Nigeria, protested over persistent power outages in the tertiary facility [50]. In Nigeria, students are uncertain about when their education will be completed owing to persistent university strikes, which further fuel frustration and the desire to pursue a normal life elsewhere (See [14]). Federal government workers are often unsure of when their salaries will be paid; it could be on the last day of the month or the first few days of the new month, but they are not certain about when it will be paid, making the system unpredictable and difficult for people to plan [6, 12]. In addition, what is normal and commonsensical is for the government and employers to adjust wages and salaries in line with the inflation rates. This is not the case in Nigeria. Strike actions are often undertaken by workers to force the government to increase salaries.

Our research has some limitations. First, the use of a non‐probability online sampling strategy limits the generalisability of the findings to all Nigerian healthcare workers. Second, self‐selection bias may have led to an overrepresentation of individuals with stronger migration intentions. Third, the cross‐sectional design prevents causal inference. Finally, stated willingness to accept occupational downgrading may not necessarily reflect actual post‐migration employment outcomes.

## 6 Conclusion

This study has demonstrated that while emigration (intention) increases in Nigeria, the country may continue to lose its HCWs to other countries and sectors within and outside the country, as most HCWs expressed willingness to leave the health sector. Nigerian HCWs may be aware of the occupational downgrading experienced by migrants abroad, yet they still prefer this option to their economic and social situation in Nigeria. The willingness to accept occupational downgrading reflects the significant global south‐north disparities in wages, security of life and property, working conditions, and the stability and predictability of the economy [6, 12]. As a result, healthcare workers opt to migrate and take on less skilled positions in the West to enjoy a better overall quality of life rather than stay in Nigeria, where they face challenging working conditions, an ever‐increasing inflation rate, and stagnant salaries. Thus, for Nigerian migrants, the decision to accept occupational downgrading post‐migration may be driven by motivations beyond just economic factors.

Pharmacists are more likely to leave the health sector if they cannot find jobs after migration, which may be connected to the experience of poor working conditions. It may also signal the existence of occupational stratification in the health sector in Nigeria. Male HCWs are more likely to leave the health sector than their female counterparts after migration, which reinforces the social norms that assign the role of financial provider to men. Improved job satisfaction in the health sector may reduce the potential loss of HCWs to other sectors within and outside Nigeria.

## Author Contributions

O.B. and T.A. conceived and designed the study and developed the questionnaire. O.B. and T.A. led data collection. O.B. and T.A. wrote and edited the manuscript. O.A. and F.B. supervised the research process. All authors read, provided input, and agreed to this version of the manuscript.

## Ethics Statement

The study was approved by the Sokoto State Ministry of Health (SKHREC/019/2023), Nigeria. The online survey included a clear introduction and consent page, where respondents who were potentially interested agreed to participate in the study before answering the survey questions. The data collected from respondents were treated anonymously and confidentially.

## Data Availability

The data that support the findings of this study are available on request from the corresponding author. The data are not publicly available due to privacy or ethical restrictions.
